# An Extremely Uncommon Case of an Internal Thoracic Vein Aneurysm

**DOI:** 10.70352/scrj.cr.25-0347

**Published:** 2025-09-20

**Authors:** Ryo Karita, Yuki Onozato, Yuki Hirai, Toshiko Kamata, Hajime Tamura, Hironobu Wada, Takashi Anayama, Ichiro Yoshino, Shigetoshi Yoshida

**Affiliations:** 1 Department of Thoracic Surgery, International University of Health and Welfare Narita Hospital, Narita, Chiba, Japan; 2 Department of Thoracic Surgery, International University of Health and Welfare School of Medicine, Narita, Chiba, Japan

**Keywords:** venous aneurysm, internal thoracic vein, video-assisted thoracic surgery

## Abstract

**INTRODUCTION:**

Venous aneurysms in the anterior mediastinum are rare. While there are scattered reports on thoracic venous aneurysms, such as those involving the azygos or brachiocephalic veins, those involving the internal thoracic vein are exceedingly rare. We herein report the case of an internal thoracic venous aneurysm that was initially suspected of being a tumor and diagnosed intraoperatively.

**CASE PRESENTATION:**

A 50-year-old woman underwent CT during hospitalization for cerebral infarction, which revealed a well-defined 14-mm nodular lesion in the anterior mediastinum. Contrast-enhanced CT performed 4 months later showed that the anterior mediastinal lesion had increased to 16 mm and exhibited slight contrast enhancement. ^18^F-fluorodeoxyglucose PET/CT demonstrated only a slight uptake in the anterior mediastinal lesion with no abnormal uptake elsewhere. Thymic epithelial tumors and cysts were included in the differential diagnosis, and video-assisted thoracoscopic surgery was planned. Intraoperative findings revealed a dark-red nodule beneath the mediastinal pleura. The right internal thoracic vein was observed to flow into the nodule and an outflow vessel draining into the right brachiocephalic vein was identified. Based on these findings, the lesion was diagnosed as an internal thoracic venous aneurysm. The right internal thoracic vein and outflow vessel were ligated, and the nodule was resected. A histopathological examination confirmed the diagnosis of venous aneurysm.

**CONCLUSIONS:**

Although extremely rare, venous aneurysms can occur in the internal thoracic vein. This condition should be considered in the differential diagnosis of enhancing solid nodules of the anterior mediastinum.

## Abbreviations

AChR
: acetylcholine receptor
CE-CT
: contrast-enhanced CT
CO_2_
: carbon dioxide
CYFRA
: cytokeratin 19 fragment
DVT
: deep venous thrombosis
FDG-PET/CT
: ^18^F-fluorodeoxyglucose-PET/CT
VATS
: video-assisted thoracoscopic surgery

## INTRODUCTION

Nodular mediastinal lesions are commonly encountered in routine clinical practice. Although thymic epithelial and germ cell tumors are the most frequently resected mediastinal lesions,^1)^ surgical resection may also be performed for vascular aneurysms other than aortic aneurysms. Reported vascular aneurysms located in the mediastinum include aneurysms of the azygos vein, superior vena cava, and brachiocephalic vein.^2–7)^ Venous aneurysms are occasionally treated surgically because of the potential risk of rupture or thrombus formation as the aneurysm enlarges.^2,3,6)^ The internal thoracic veins are a pair of vessels that receive blood from the chest wall and mammary glands and drain into the brachiocephalic veins. They are relatively small in diameter, and only 2 cases of venous aneurysms of the internal thoracic vein have been reported to date.^8,9)^

We herein report a surgically treated case of an internal thoracic venous aneurysm that was incidentally detected on CT.

## CASE PRESENTATION

**Fig. 1:**
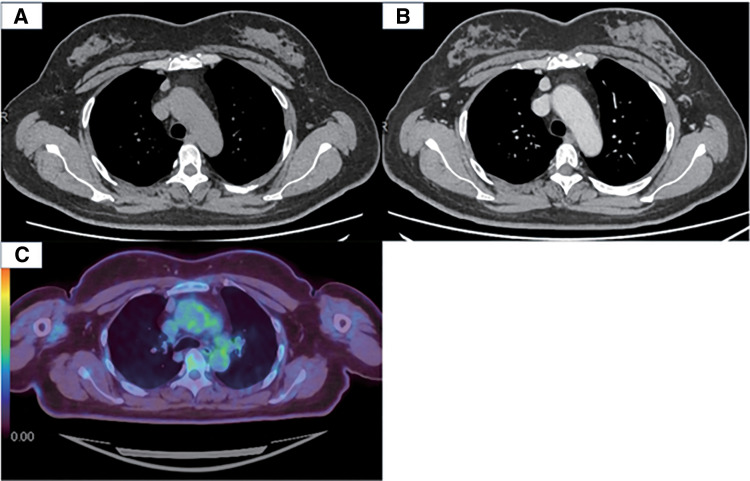
Preoperative chest imaging. (**A**) CT showed a well-defined, smooth nodule measuring 14 mm on the right side of the anterior mediastinum. (**B**) Four months later, contrast-enhanced CT showed that the nodule had increased in size to 16 mm. (**C**) ^18^F-fluorodeoxyglucose-PET/CT showing only a slight ^18^F-fluorodeoxyglucose uptake in the nodular lesion.

A 50-year-old woman was referred to our department of thoracic surgery for the further evaluation and treatment of a nodular lesion in the anterior mediastinum. She had been hospitalized in our department of neurosurgery 5 months earlier for a left pontine infarction. During hospitalization, chest CT revealed a well-defined smooth-surfaced 14-mm nodule on the right side of the anterior mediastinum. CE-CT performed 4 months later showed that the nodule had increased in size to 16 mm. The nodule was connected to the right internal thoracic vein (**Fig. 1**). The patient had a 13-pack-year smoking history and had quit smoking at 46 years old. She had comorbid hypertension, diabetes mellitus, and dyslipidemia for which she was receiving medical treatment. Chest radiography revealed no abnormalities. Blood tests revealed a mildly elevated CYFRA level of 2.9 ng/mL. Tests for autoimmune antibodies, including anti- AChR antibodies, were all negative. FDG-PET/CT showed a minimal FDG uptake in the nodular lesion (**Fig. 1**). The differential diagnoses included thymic epithelial tumors and thymic cysts; while typical thymic cysts rarely show enhancement, we considered thymic cysts with a coexisting thymoma or hemorrhage as a differential diagnosis because they can be associated with enhancement. Given the patient’s preference, surgery was performed for diagnostic and therapeutic purposes.

**Fig. 2:**
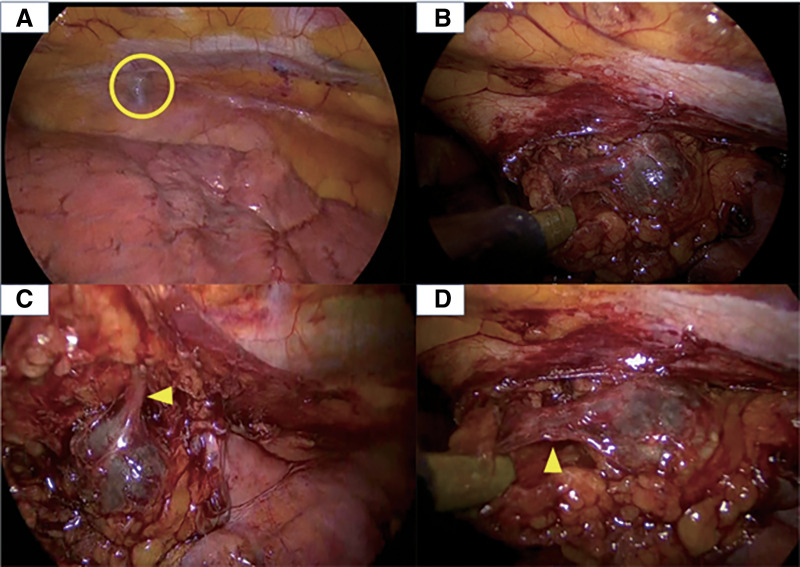
Intraoperative findings. (**A**) A dark-red nodule was visible beneath the mediastinal pleura (yellow circle). (**B**) The entire anterior mediastinal tumor. (**C**) The inflow from the right internal thoracic vein (yellow arrowhead). (**D**) The outflow into the brachiocephalic vein (yellow arrowhead).

VATS via the right thoracic approach was performed using 3 ports and CO_2_ insufflation. No pleural effusion or intrathoracic adhesions were observed. A dark-red nodule was visible beneath the mediastinal pleura. To maintain a safe distance from the phrenic nerve, the mediastinal pleura was incised along the margin of the nodule to allow for full visualization of the lesion. As demonstrated on preoperative imaging, the nodule was connected to the right internal thoracic vein and was smoothly continuous with it. Intraoperatively, the nodule was identified as a venous aneurysm of the internal thoracic vein (**Fig. 2**). The inflow and outflow vessels were divided, and the aneurysm was carefully dissected from the pericardium and completely resected. Histopathological examination revealed a large vein embedded in fatty tissue, with an irregularly thickened wall, leading to a diagnosis of venous aneurysm. Malignant features and any thrombus were not observed. The postoperative course was uneventful, and the patient was discharged on POD 4. She is currently being followed up on an outpatient basis, and chest CT performed 3 months after surgery showed no evidence of recurrence.

## DISCUSSION

This report describes a surgically treated case of an internal thoracic venous aneurysm incidentally detected on chest CT performed during hospitalization for an unrelated condition. As preoperative examinations could not rule out the possibility of malignancy, surgery was performed for both diagnostic and therapeutic purposes, leading to a diagnosis of an internal thoracic venous aneurysm.

To our knowledge, only 2 cases of internal thoracic vein aneurysms have been reported. Almassi reported a surgical case in a 52-year-old woman, which was the first report concerning an internal thoracic vein aneurysm.^8)^ The patient had experienced a traffic accident 16 months prior and underwent fixation surgery for a right leg fracture. One week later, she was readmitted with a sudden onset of shortness of breath and wheezing. CE-CT ruled out pulmonary embolism but revealed a 22-mm anterior mediastinal lesion. A similar lesion had been noted on CT at the time of the traffic accident but had increased in size over 16 months. Surgery via an upper sternal split was performed, leading to the diagnosis of internal thoracic vein aneurysm. Petrella et al. reported a case of internal thoracic vein aneurysm in a 45-year-old woman.^9)^ During follow-up after surgery for malignant melanoma, a 40-mm anterior mediastinal lesion was detected on CT. FDG-PET/CT showed a moderate FDG uptake in the anterior mediastinal lesion. Thymoma was suspected; however, considering the history of malignant melanoma surgery, a CT-guided biopsy was planned. Pre-biopsy CE-CT showed that the anterior mediastinal lesion exhibited contrast enhancement similar to the superior vena cava in the venous phase and had continuity with the internal thoracic vein, leading to the diagnosis of an internal thoracic vein aneurysm. The patient was asymptomatic and under observation. In our case, the patient was asymptomatic, and FDG-PET/CT showed only a slight uptake in the lesion; however, considering the increasing size, surgical treatment was chosen. In cases of increasing size on CT or in Petrella et al.’s case, wherein the aneurysm showed an uptake on PET despite being a vascular lesion, differentiation from thymic epithelial tumors, which are more common, is difficult if this disease is not considered. Because the lesion is enhanced to a similar degree as the blood vessels on CE-CT, careful attention is required to diagnose strongly enhancing nodules. Dynamic contrast-enhanced CT is widely used to evaluate abdominal hemangiomas, and its application to mediastinal hemangiomas has also been reported. Cheung et al. reported that dynamic CT was useful for the preoperative diagnosis of a superior mediastinal cavernous hemangioma in a young male.^10)^ In their report, they noted that although early scans were not diagnostic, the delayed washout of the contrast medium was a significant clue for suspecting the tumor. Li et al. reported on a cavernous hemangioma that extended from the neck to the anterior mediastinum.^11)^ They found that dynamic CT showed peripheral nodular enhancement in the early phase and a gradual centripetal fill-in pattern in the delayed phase. These reports suggest that dynamic CT may also be a valuable tool for diagnosing lesions that show a contrast enhancement effect. The preoperative diagnosis might have been possible in this case as well if dynamic CT had been performed.

Venous aneurysms are rare vascular malformations, 1st reported by Osler in 1913.^12)^ Teter et al. summarized 508 reported cases of venous aneurysms up to 2017, of which 42 cases (8.3%) were thoracic venous aneurysms.^13)^ Symptoms associated with thoracic venous aneurysms typically occur in cases where thrombus formation leads to pulmonary embolism,^2)^ rupture of the aneurysm,^14)^ or an enlarged venous aneurysm compresses or irritates adjacent structures.^3)^ Usually, the risk of symptoms increases with enlargement of the aneurysm, and small aneurysms are unlikely to be discovered because of these symptoms. Among thoracic venous aneurysms, azygos vein aneurysms are frequently reported. Yao et al. reported 73 cases of azygos vein aneurysm. Of these, chest pain occurred in 13 cases, chest tightness and dyspnea in 10, cough in 4, and dysphagia in 3.^4)^ The internal thoracic vein was smaller in diameter than the azygos vein and was not closely associated with major adjacent structures. Therefore, venous aneurysms of the internal thoracic vein may be less likely to produce symptoms than other thoracic venous aneurysms. The causes of venous aneurysms are classified as idiopathic, traumatic, or congenital due to connective tissue disease.^15)^ In this case, there was no history of trauma or signs suggesting a connective tissue disorder, leading to the conclusion that the venous aneurysm was idiopathic.

There are currently no established guidelines for the treatment of mediastinal venous aneurysms. Although complications associated with venous aneurysms are rare, cases of aneurysmal rupture, thrombosis, and acute pulmonary thromboembolism have been reported.^2–4)^ In large azygos vein aneurysms, slow blood flow may predispose individuals to thrombus formation, and some reports advocate early surgical intervention. However, clear thresholds regarding the diameter or growth rate of aneurysms associated with thrombus formation remain unclear. Conversely, considering the mortality associated with surgery, some reports recommend conservative management with anticoagulation therapy for thromboprophylaxis.^2–9,12–14)^ Surgical consideration is reported for cases with compression of adjacent organs, occurrence of clinical symptoms, thrombus formation during anticoagulation therapy, contraindications to anticoagulation therapy, concurrent embolism, enlargement of aneurysm diameter, and presence of connective tissue disease.^2,3)^ Although there are reports of endovascular treatment for popliteal arteriovenous fistulae^16)^ and azygos vein aneurysms,^4)^ we could not find any reports of endovascular treatment for a small mediastinal venous aneurysm like the 1 in this case. Given that a definitive diagnosis had not been obtained and catheter access was considered difficult, we opted for surgery in this case. In the 2 previous case reports of this disease,^8,9)^ the management was either surgical resection or observation, and the outcomes of either approach are unclear; therefore, the most appropriate treatment and the prognosis for internal thoracic vein aneurysms remain unknown. In the present case, the patient had a good outcome with no symptoms or sequelae at 6 months postoperatively.

## CONCLUSIONS

We encountered a surgical case of internal thoracic vein aneurysm. To our knowledge, only 2 other cases of this disease have been reported, and many aspects regarding established treatment methods and the prognosis remain unclear. Continued follow-up is important, and we hope for the accumulation of more reports and the development of knowledge regarding this disease in the future.

## DECLARATIONS

### Availability of data and materials

Data sharing is not applicable to this article because the datasets were neither generated nor analyzed in the case report.
